# Bond strength to dentin after chemomechanical caries removal

**DOI:** 10.4317/jced.62886

**Published:** 2025-09-01

**Authors:** Danúbia Matos, Fernanda de Castro, Fernanda Rocha, Ivan Barreiros, Bruna Genari, Francisca Jardilino, Monica Yamauti, Célia Lanza

**Affiliations:** 1 Department of Clinical Dentistry, Pathology and Oral Surgery, Faculty of Dentistry, Federal University of Minas Gerais /UFMG

## Abstract

**Background:**

This study aimed to evaluate bond strength of self-etching adhesive to dentin following chemomechanical dentin or burs carious removal.

**Material and Methods:**

Twenty-two sound molars were sectioned transversely to achieve complete exposure of dentin, followed by the induction of artificial caries. The teeth were randomly divided into two groups: Brix—carious dentin removal with papain-based gel (Brix 3000), and Burs—carious dentin removal with drills. A morphological analysis of prepared dentin was performed on two samples from each experimental group using scanning electron microscopy. Teeth were restored using a self-etch adhesive system (Clearfil SE Bond, Kuraray) and composite resin (Filtek Z350 XT, 3M Oral Care). For the microtensile bond strength μTBS test, beams were tested under tensile stress after 24 hours of storage in distilled water. Fractographic failure mode was performed using a stereomicroscope and two beams from each group were analyzed using scanning electron microscopy (SEM). The data were analyzed using an independent samples t-test with a significance level of α=0.05.

**Results:**

The μTBS ranged from 23.84 ± 5.77 MPa for the Brix group to 28.91 ± 4.82 MPa for the burs group. There was no statistical difference between the groups (*p* = 0.06). The adhesive failure was the most prevalent in both groups.

**Conclusions:**

The chemomechanical carious dentin removal using papain gel formulation Brix3000® did not affect bond strength compared to bur removal method.

** Key words:**Dental caries, Tensile strength, Dental adhesive.

## Introduction

Dental caries remains the most prevalent chronic disease in childhood, with a global prevalence of 35% across all ages in permanent dentition, despite a significant reduction in caries prevalence in several countries [1]. Dental caries is a biofilm-mediated and diet-modulated disease that results in the dissolution of minerals from dental hard tissues [1]. The onset and progression of caries lesions are dynamic processes, occurring when episodes of demineralization (predominance of risk factors) exceed those of remineralization (predominance of protective factors). Once dentin is involved, the reversal of mineral loss is no longer possible, and restorative treatment becomes necessary [1].

Among the methods of caries lesion removal, the conventional technique using rotating instruments (burs) is the oldest and most employed today. This method is associated with excessive extension into dental tissue, leading to a higher risk of pulp exposure [2]. Additionally, patients often perceive it as painful, unpleasant, and anxiety-inducing [3,4]. To address these drawbacks, minimally invasive methods-such as air abrasion, atraumatic restorative treatment (ART), sonic abrasion, and chemomechanical caries removal-have been proposed to enhance tissue preservation and improve patient comfort [4,5].

Chemomechanical caries removal methods are a proven effective alternative to the conventional approach [2,5,6]. Although there is variation among the categories of chemomechanical products, this technique generally employs substances that denature collagen fibrils and partially degrade and dissolve necrotic dentin. This facilitates the removal of infected tissue using blunt hand instruments while allowing for the preservation of affected tissue that may be subject to remineralization [3,4,6,7]. However, variations in the formulation or processing of these materials can impact adhesion to dental tissues.

Chemomechanical caries removal can be achieved using products based on sodium hypochlorite or enzymes [5]. While sodium hypochlorite-based products are effective in removing carious tissue, the come with high technical sensitivity, cost, and longer application times [5,6]. In contrast, enzyme-based options like Brix3000® utilize emulsion buffer encapsulating (EBE) technology, which enhances papain concentration (3000 U/mg every 10%), stability, and optimal pH [6]. This formulation allows for more effective proteolysis of carious tissue, better antimicrobial properties, and less sensitivity to storage conditions, all while preserving adjacent living tissues, mucosa, and healthy dentin [3,6]. While the efficacy of chemomechanical methods for caries lesion removal is well established in the literature, there are still limited studies evaluating Brix 3000® and its effect on the adhesion of restorative materials, underscoring the need for further studies.

Therefore, the aim of this study was to evaluate bond strength following chemomechanical dentin carious removal compared to the conventional method. The null hypothesis tested was that there would be no difference in bond strength, regardless of the caries removal method utilized.

## Material and Methods

All ethical precepts related to research involving human beings were respected (CAAE 15912719.1.0000.5149).

The composition and materials used in the study are shown in  Table 1.

1. Tooth preparation 

For this study, 22 sound teeth were used. The sample size calculation was conducted using the USP Bauru Sample Size Calculator (University of São Paulo, Bauru, São Paulo, Brazil), employing a 95% confidence interval and 80% study power, along with data from Cechin *et al*., 2010 [8]. The roots of the teeth were embedded in acrylic resin (VIPI Flash, VIPI, Odontológicos, Pirassununga, SP, Brazil), and the occlusal surfaces were sectioned to remove the cusps, fully exposing the mid-coronal dentin using a precision metallographic cutter (Isomet 1000, Buehler, Lake Bluff, IL, USA). A layer of epoxy adhesive (Araldite Hobby, TekBond Saint-Gobain, Cotia, SP, Brazil) was applied to the side walls to ensure that only the dentin surface was exposed to artificial caries development.

2. Preparation of the cariogenic solution and caries induction

The teeth were then subjected to artificial caries induction following the microbiological methodology described by Lenzi [9]. The cariogenic medium was prepared with 3.7 g de brain heart infusion (BHI) (Sigma-Aldrich, St Louis, Missouri, EUA), 0.5 g of yeast extract (Neogen, Lansing, Michigan, USA), 1 g of glucose (Êxodo Científica, Sumaré, SP, Brazil), and 2 g of sucrose (Êxodo Científica). This solution was sterilized in an autoclave at 121°C for 15 minutes. Strains of *Streptococcus mutans* (INCQS 00446), ajusted to an optical density of 0.5 using the McFarland standard (Laborclin,Pinhais, Paraná, Brazil) corresponding to 1.5 x 108 CFU/mL were added to the cariogenic solution. Immediately after the addition of *Streptococcus mutans* strains to the cariogenic solution, the teeth were immersed in the solution and incubated at 37°C in a microaerophilic jar (JAO 401, Permution, E.J Krieger & Cia Ltda, Curitiba, PR, Brazil). The total contact period between the dentin and the cariogenic solution lasted 14 days; during which the solution was changed, and a new inoculation of microorganisms was performed every 48 hours. The detection of cavities through tactile and visual assessment was performed by a single blinded evaluator. The dentin was slightly darkened, was softened to touch with an exploratory probe, and could be removed using a cutting hand instrument.

3. Caries removal

After the caries induction process, the teeth were randomly divided into two groups using the =RANDOM function in Microsoft Excel (Microsoft Corporation, Redmond, WA, USA), as detailed below:

Brix—chemomechanical caries removal using Brix 3000 papain gel and a non-cutting instrument, followed by composite resin restoration.

Burs—mechanical caries removal using a rotary instrument (slow-speed stainless-steel drill), followed by composite resin restoration.

For chemomechanical caries removal, a 10% papain gel with an enzymatic concentration of 3,000 U/mg (BRIX 3000) was applied to the dentin, following the manufacturer’s instructions. After 2 minutes of gel application, the carious lesion (softened tissue) was removed using a blunt-edged spoon with pendulum movements, applying no pressure. This procedure was repeated until healthy dentin was reached. For mechanical caries removal, No. 4 spherical steel drills were employed at low speed. Each drill was used on up to four teeth. The resulting dentin after caries removal with a bur was hard and cut-resistant, resembling healthy dentin.

4. Bonding procedures 

The adhesive system (Clearfil SE Bond, Kuraray Noritake Dental Inc., Kurashiki, Japan) was applied following the manufacturer`s instructions. The primer was applied for 20 seconds and left undisturbed and air-dried for 5s. The bond was then applied and light-cured for 10 seconds (Radii Cal Plus, SDI, Melbourne, Australia) at 1,000 mW/cm. The resin composite (A1 color, Filtek Z350 XT, 3M Oral Care, Sumaré, SP, Brazil) was built up in increments of approximately 1.5 mm. Each increment was light-activated for 20 seconds using a light-curing unit (Radii Cal Plus, SDI). The restored tooth was subsequently stored in distilled water at 5°C for 24 hours.

5. Microtensile bond strength testing (μTBS)

The teeth were sectioned into four to ten beams (area of 1.0 mm2) with a slow-speed saw on a precision metallographic cutter (Isomet 1000 Buehler; Lake Bluff, IL, USA) under water irrigation.10 After 24 hours, the specimens were fixed to a microtensile device and tested on a mechanical testing machine (Microtensile Tester, Bisco, Inc., Shaumburg, IL, USA) at a crosshead speed of 0.5 mm/min until failure. Analysis of the fractographic failure mode was performed using a stereomicroscope (Stemi DV4, Zeiss, Germany) at 100X magnification to determine the mode of failure: adhesive (AD), mixed (MI), cohesive in dentin (CD), or cohesive in composite (CC).

6. Analysis in scanning electron microscopy (SEM)

Four teeth were designated for descriptive analysis using SEM, comprising two samples from each experimental caries removal group without restoration, as well as two beams from each group.

7. Statistical analysis 

IBM SPSS Statistics Software 27.0 (IBM Corporation, Armonk, New York, USA) was employed to analyze the bond strength data. Normality was confirmed using Shapiro-Wilk test (*p* = 0.86) and homogeneity was assessed using Levene´s test (*p* = 0.55). Subsequently, the data were examined using an independent samples t-test. The confidence level was set at α = 0.05.

## Results

**Figure 1:**
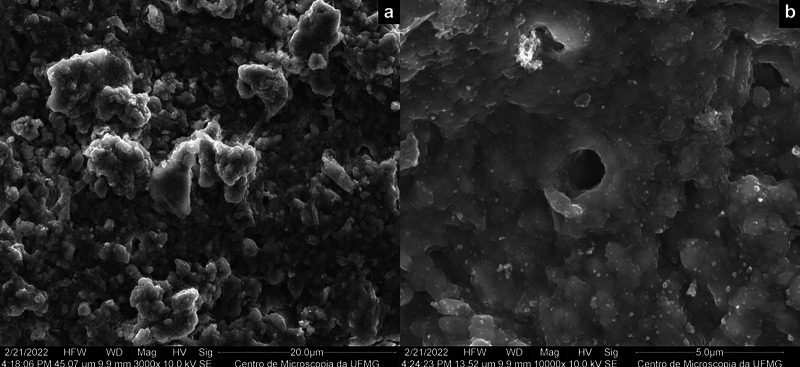
SEM micrographs showing the dentin surface obtained after caries removal with Brix 3000® (a) at lower magnification (3,000X) and (b) at higher magnification (10,000X).

**Figure 2:**
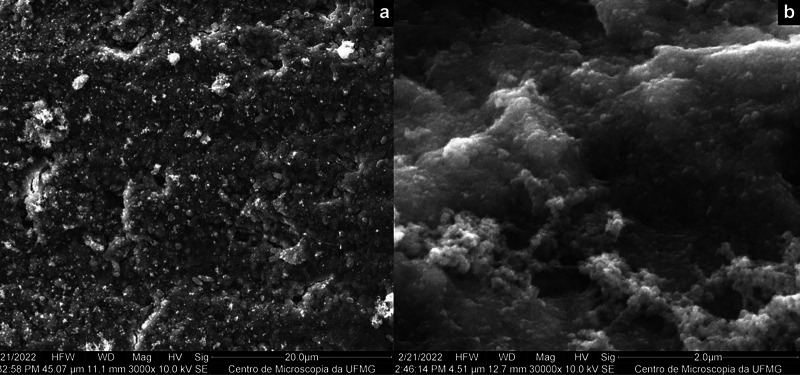
SEM micrographs showing the dentin surface obtained after the conventional method of caries removal (a) at lower magnification and (b) at higher magnification.

The remaining dentin surface following chemomechanical or conventional caries removal exhibited distinct patterns, while also displaying some similarities. After the application of Brix 3000®, the dentin appeared rough and irregular, characterized by a loose smear layer and some exposed dentinal tubules (Fig. 1). In contrast, conventional removal using a rotary instrument resulted in an irregular surface with a dense/compacted smear layer that occluded the dentinal tubules. Drill marks, including grooves and excavations, were also evident (Fig. 2).

**Figure 3:**
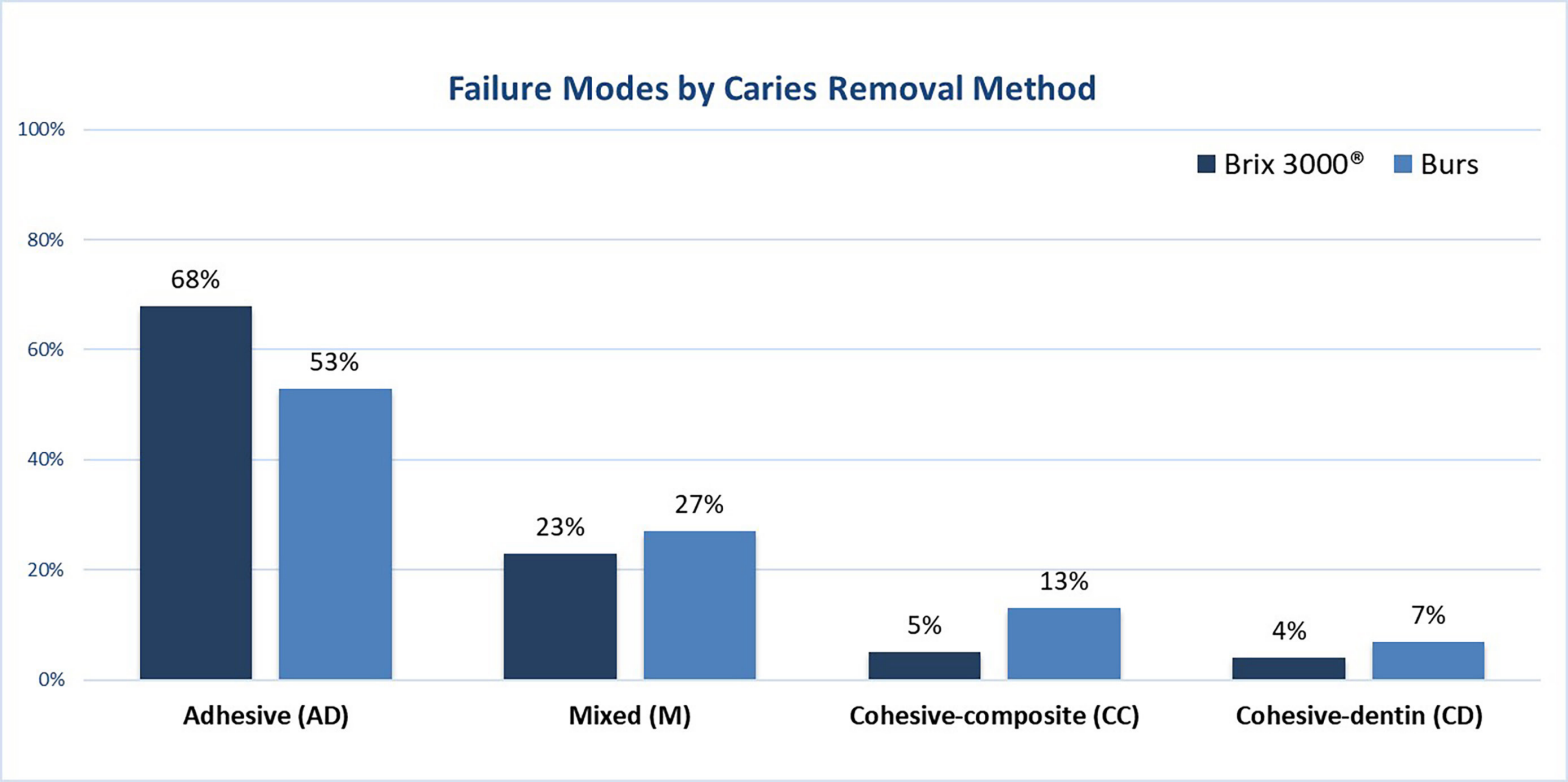
Distribution of failure modes for the two caries removal methods (Brix 3000® and burs). AD: adhesive failure; M: mixed failure; CD: cohesive failure in composite; CC: cohesive failure in dentin.

**Figure 4:**
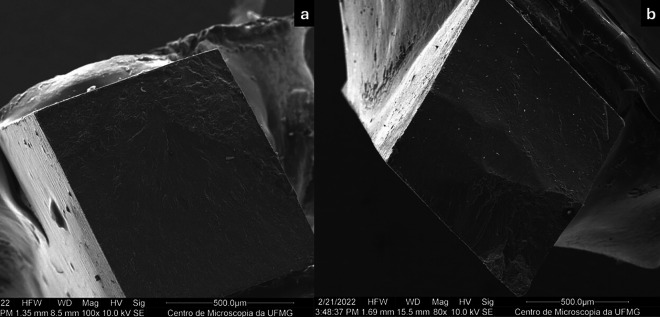
SEM micrographs showing adhesive fracture after chemomechanical removal with Brix 3000 ® (a) and bur (b).

The microtensile bond strength results and the mode of failure are shown in  Table 2 and Figure 3, respectively. The μTBS ranged from 23.84 ± 5.77 MPa for the Brix group to 28.91 ± 4.82 Mpa for the burs group. The type of caries lesion removal- chemomechanical or conventional-did not significantly influence the immediate μTBS (*p* = 0.06). Each tooth served as an experimental unit, yielding an average of 7.40 ± 3.17 and 10.33 ± 4.69 beams tested in the Brix and Burs groups, respectively. No premature failures occurred in either group. Adhesive fractures were more frequently observed in both groups (Fig. 3), confirmed by scanning electron microscopy (SEM) images (Fig. 4).

## Discussion

The tested hypothesis was accepted, as no statistical difference in bond strength was found among the different carious dentin removal methods evaluated in this study. These results corroborate previous findings in the literature [11,12], highlighting the potential of using chemomechanical caries removal in clinical situations, especially with combined with a self-etch adhesive system. This represents a significant step toward implementing chemomechanical removal, offering the benefits of less invasive and more comforTable dental treatments without concerns about bond performance [12].

The literature indicating that Brix3000® demonstrated promising results in effective carious tissue removal and safe usage [6,7,13,14] without inducing indirect effects via transdentinal diffusion or stimulating pro-inflammatory processes mediated by reactive nitrogen species (ROS) [14]. This minimally invasive method not only facilitates tissue preservation and reduces the risk of pulp exposure but also offers better patient acceptance—particularly among those who experience anxiety and pain during treatments involving rotary instruments [15-19]. These advantages underscore the relevance of chemomechanical removal techniques in pediatric dentistry, for patients with special needs, and for individuals with dental phobias.

The artificial caries induction method closely simulates clinical conditions while standardizing lesions, as dentin is affected by bacterial agents and the rate of caries development [9,20]. Among various methods, the microbiological approach effectively generates lesions that closely resemble natural ones, characterized by color changes, distinct areas, and increased softness and depth [20]. This method reproduces the morphological pattern of collagen degradation, resulting in two layers of colored carious dentin without the formation of tertiary dentin, as there is no intratubular deposition of dentin via odontoblastic activity [9,20] Furthermore, this model ensures that carious lesions are produced under consistent conditions, addressing the lack of standardization associated with natural caries lesions, which can pose technical challenges during bonding tests. Even when utilizing artificial caries, the microtensile bond strength (µTSB) values fall within the range reported in other studies that investigated natural caries [12]. Adhesion to caries-affected dentin presents challenges due to lower mineral content surrounding collagen fibrils, occluded dentinal tubules, increased demineralization and porosity, moisture presence, and a smear layer containing acid-resistant minerals [11,21,22]. These factors can negatively impact the hybridization of resin to dentin, resulting in lower bond strength results.

Previous studies [11,12] that evaluated rotary instruments and chemomechanical caries reported bond strength results similar to those found in this study. Despite some differences between the studies, particularly in adhesive systems and natural caries, the results were similar and corroborate our findings. The etch-and-rinse adhesive system, which involves phosphoric-acid etching, was the most affected by the caries removal technique and less preferred for treating dentin [23]. The present SEM images showed a compacted smear layer that occluded the dentinal tubules for rotary instrument caries removal, which is not observed with chemomechanical removal. Previous studies characterized chemomechanical removal, similarly, noting areas with an amorphous layer resembling the smear layer, along with areas where these tubules are completely exposed [24,25]. The absence of a dense smear layer occluding dentinal tubules in chemomechanical removal may suggest a beneficial combination of chemomechanical techniques and self-etch adhesive system. This approach modifies rather than completely removes the smear layer, resulting in the formation of smear tags. Additionally, when using a self-etch system on a dentin substrate, there is the advantage of having fewer exposed collagen fibrils subjected to enzymatic degradation, along with chemical interaction in addition to micromechanical interlocking and consequently favor longitudinal bond strength [26] With ‘mild’ (pH ≈ 2) self-etch adhesives, a submicron hybrid layer containing substantial hydroxyapatite (HAp) crystals that protect the collagen fibrils is typically observed. The resulting micromechanical interlocking through submicron hybridization is complemented by a primary ionic interaction between the residual HAp and functional monomers such as 10-MDP (10-methacryloyloxydecyl dihydrogen phosphate), present in Clearfil SE Bond, which has shown to be sTable against degradation. The stronger the chemical interaction potential, the less soluble the resulting calcium salts become. Two-step self-etch adhesives allow for the application of a separate hydrophobic adhesive resin following the hydrophilic self-etch primer. This results in a more hydrophobic interface, which also contributes to enhanced bond durability [26].

One limitation of this study is that the longevity of bond strength was not evaluated. Although immediate bond strength is commonly assessed, it is recommended that the bonding effectiveness of adhesives also be evaluated under clinical conditions or through aging simulations [27]. Among the available methods, water storage and thermocycling are considered the most relevant for simulating aging. The storage of micro-specimens in water for three months has demonstrated significant mechanical and morphological evidence of degradation, resembling *in vivo* aging [28]. A short regimen of 500 cycles, as recommended by the ISO TR 11450 standard, has proven insufficient as an efficient aging protocol, with approximately 100,000 cycles being necessary [29]. Bond strength data obtained after *in vitro* aging procedures have been shown to reliably predict long-term clinical performance, particularly over periods starting from five years [27].

In addition to potential implications for long-term durability, although the bond strength comparison between the Brix3000® and Burs groups did not reach statistical significance according to the conventional *p-value* threshold, the results may still hold clinical relevance. To complement the interpretation of statistical significance, the effect size was calculated, yielding a Cohen’s d of 0.95, which indicates a large effect. This suggests that, from a clinical perspective, the burs protocol may lead to improved bond strength compared to the Brix method, even in the absence of statistically significant differences. This finding may be further supported by future studies evaluating long-term performance.

The demineralized collagen fibrils infiltrated by adhesive resin resulted in a polymeric matrix surrounding the collagen, as well as areas of non-protected dentin. Both components are susceptible to failure—the polymeric matrix and exposed collagen fibrils—leading to *in vivo* adhesive failure patterns [30,31]. A similar fracture pattern was observed in the present study and in previous research, even with sound dentin [11,21,32]. The concentration of stress resulted in fractures at the interface for all groups, indicating the failure in the union between the collagen fibrils and the polymeric matrix within the hybrid layer. The similarity of failure patterns between groups aligns with the absence of differences in the thickness of the hybrid layer and the gap formation at the tooth-resin adhesive interface for the different caries removal methods—Brix 3000® and rotary instrument [25].

## Conclusions

Within the limitations of this study, it was possible to conclude that chemomechanical carious dentin removal using papain gel formulation Brix3000® did not affect bond strength compared to conventional removal method. However, further *in vitro* studies evaluating the long-term durability of the bond, as well as clinical studies, are warranted to provide a more comprehensive assessment of Brix 3000®.

## Figures and Tables

**Table 1:** Materials used in this study.

Brand name	Composition	Manufacturer
Brix 3000®	Components: papain 30,000 U/mg, excipients 10 g (propylene glycol, citrus pectin, triethanolamine, sorbitan monolaurate, disodium phosphate, monopotassium phosphate, toluidine blue, 100 mL distilled water q.s.)	BRIX S.R.L., CarcaraÃ±Ã¡, Province of Santa Fe, Argentina.
*Filtek Z350 XT	Monomers: Bis-GMA, UDMA, TEGDMA, PEGDMA, and Bis-EMA Filler particles: zirconia/silica Other components: catalysts, stabilizers, and pigments	3M Oral Care, SumarÃ©, SP, Brazil
Clearfil SE Primer (self-etching)	Main components: 10-methacryloxydecyl dihydrogen phosphate (MDP), 2-hydroxyethyl methacrylate (HEMA), hydrophilic dimethacrylate, camphorquinone, N,N-diethanol-p-toluidine, water	Kuraray Noritake Dental Inc., Okayama, Japan
Clearfil SE Bond Adhesive	Main components: 10-ethacryloxydecyl dihydrogen phosphate (mdp), bis-phenol a glycidyl dimethacrylate (Bis-GMA), 2-hydroxyethyl methacrylate (HEMA), hydrophobic dimethacrylate, camphorquinone, N,N-diethanol-p-toluidine, silanized colloidal silica	Kuraray Noritake Dental Inc., Okayama, Japan

**Table 2:** Microtensile bond strength (µTBS) results and number of beams per tooth are expressed in MPa as mean ± SD.

	µTBS	Number of beams per tooth	Premature failure	Normality Sig.	Homogeneity Sig.	Sig. (2-tailed)
Brix	23.84 ± 5.77A	7,40 ± 3,17	0	0.863	0.549	0.060
Burs	28.91 ± 4.82A	10,33 ± 4,69	0	0.187		

## Data Availability

The datasets used and/or analyzed during the current study are available from the corresponding author.
